# Sudden cardiac death in early repolarization syndrome despite detailed evaluation for syncope: a case report

**DOI:** 10.1093/ehjcr/ytag663

**Published:** 2026-09-10

**Authors:** Tsubasa Saeki, Soichiro Yamashita, Masanori Okuda

**Affiliations:** Department of Cardiology, Hyogo Prefectural Awaji Medical Center, 1-1-137 Shioya, Sumoto City 656-0021, Japan; Department of Cardiology, Hyogo Prefectural Awaji Medical Center, 1-1-137 Shioya, Sumoto City 656-0021, Japan; Department of Cardiology, Hyogo Prefectural Awaji Medical Center, 1-1-137 Shioya, Sumoto City 656-0021, Japan

**Keywords:** Early repolarization syndrome, Sudden cardiac death, Insertable cardiac monitor, Ventricular fibrillation, Risk stratification, Case report

## Abstract

**Background:**

Early repolarization syndrome (ERS) has typically been regarded as a benign electrocardiographic variant; however, recent studies have demonstrated its association with ventricular fibrillation (VF) and sudden cardiac death (SCD) in specific patients. Risk stratification poses significant challenges due to the high prevalence of early repolarization patterns in healthy individuals and the low incidence of malignant arrhythmias.

**Case summary:**

We report a case of a 30-year-old man with no significant medical history who presented with unexplained syncope and demonstrated J-wave abnormalities in the lateral leads. Since the patient did not meet guideline-based criteria for implantable cardioverter-defibrillator (ICD) implantation, he underwent management with an insertable cardiac monitor (ICM) for long-term rhythm surveillance. Six months later, the patient suffered SCD secondary to VF, which the ICM confirmed.

**Discussion:**

This retrospective review revealed several subtle indicators of arrhythmic risk, including nocturnal J-wave augmentation and pilsicainide-induced J-point elevation. This case highlights the limitations of current ERS risk-stratification strategies and underscores the need to consider non-ICD preventive approaches, such as enhanced risk communication and strategic installation of an automated external defibrillator for patients who do not meet ICD therapy criteria but remain at potential arrhythmic risk.

Learning points

- The pilsicainide challenge test induced J-point elevation in lead V1, suggesting potential utility as a predictor of ventricular tachycardia/ventricular fibrillation.
- Individualized risk communication is critical when managing early repolarization syndrome patients who present with subtle risk markers.

## Introduction

Early repolarization (ER), traditionally considered a benign electrocardiogram (ECG) variant, has been associated with ventricular fibrillation (VF) and sudden cardiac death (SCD) in selected populations.^1,2^ Although ER patterns are common in healthy young adults, particularly men, identifying high-risk individuals remains difficult because malignant events are rare.^3^ Current guidelines limit implantable cardioverter-defibrillator (ICD) therapy to patients with documented VF or polymorphic ventricular tachycardia (VT), excluding those with isolated J-waves or syncope.^4^ We report a young man with ER syndrome who developed SCD after an initial syncopal episode despite insertable cardiac monitor (ICM) surveillance and low-risk classification. This case highlights limitations of current risk stratification and suggests broader preventive strategies, including patient education and emergency preparedness.

## Summary figure

**Figure:**
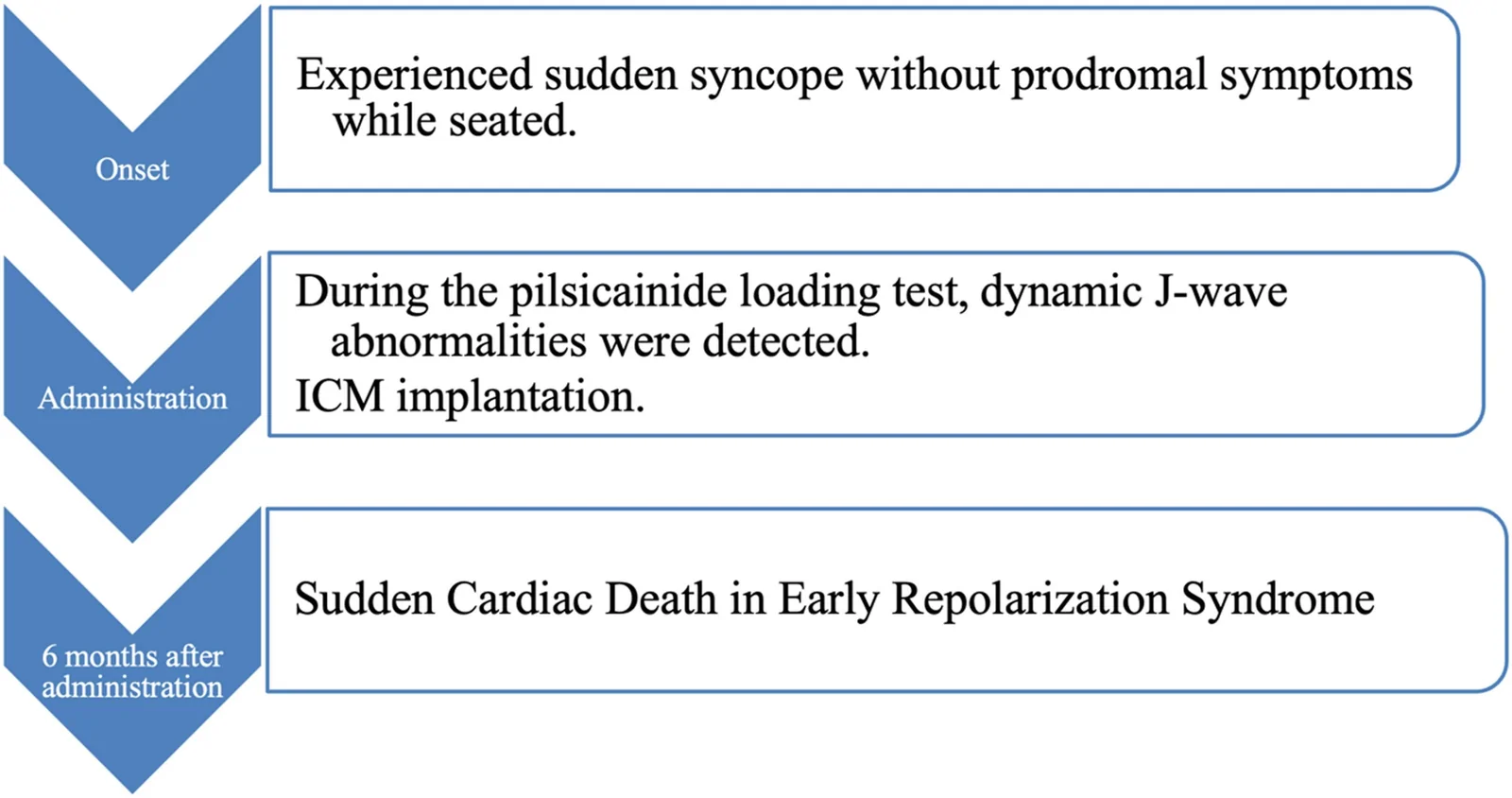


## Case presentation

**Figure 1:**
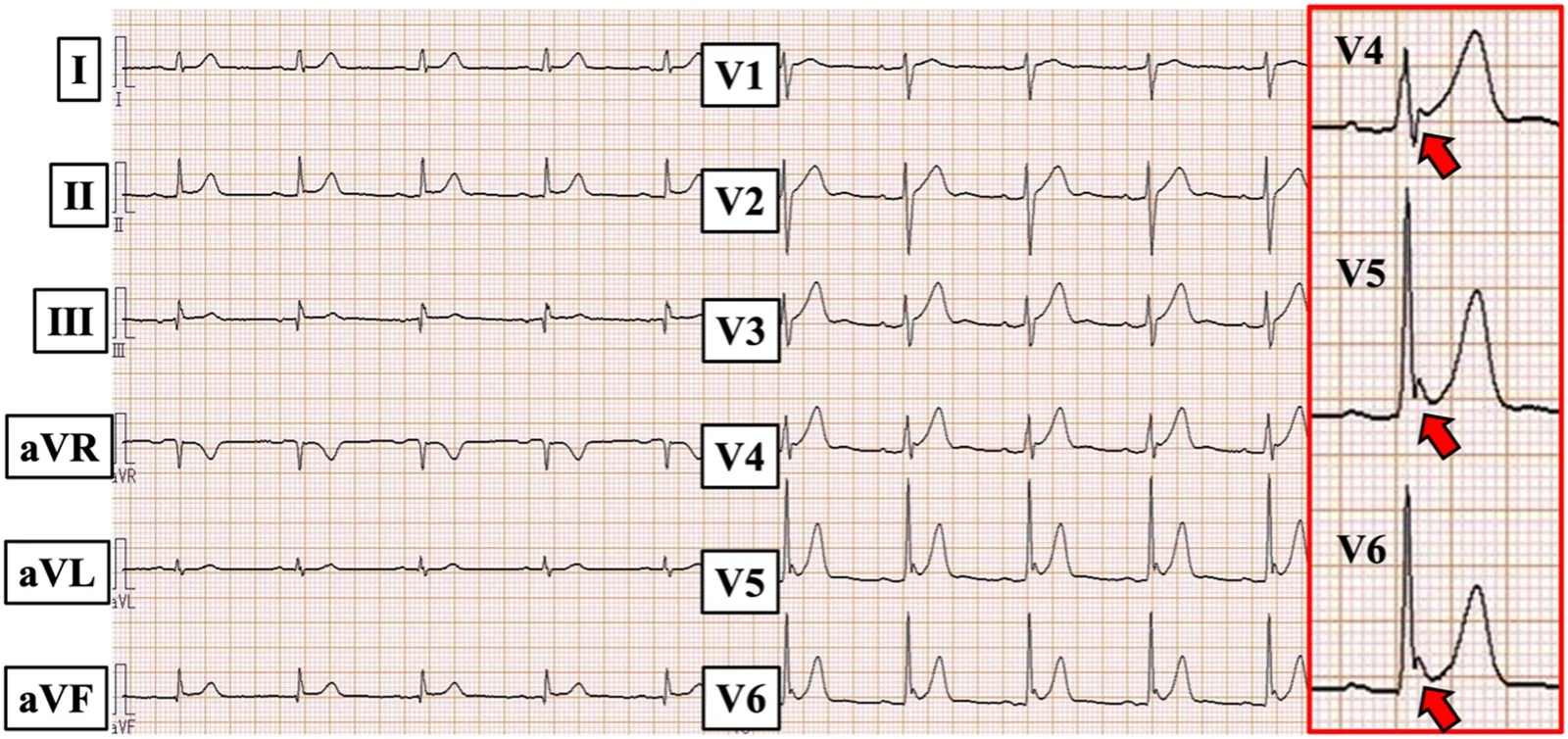
Twelve-lead electrocardiogram at the time of admission. Electrocardiogram revealed 0.2 mV J-waves in leads V4 through V6, without Brugada pattern or QT prolongation. Electrocardiograms were recorded at a paper speed of 25 mm/s and a calibration of 10 mm/mV with baseline drift filtering.

**Figure 2:**
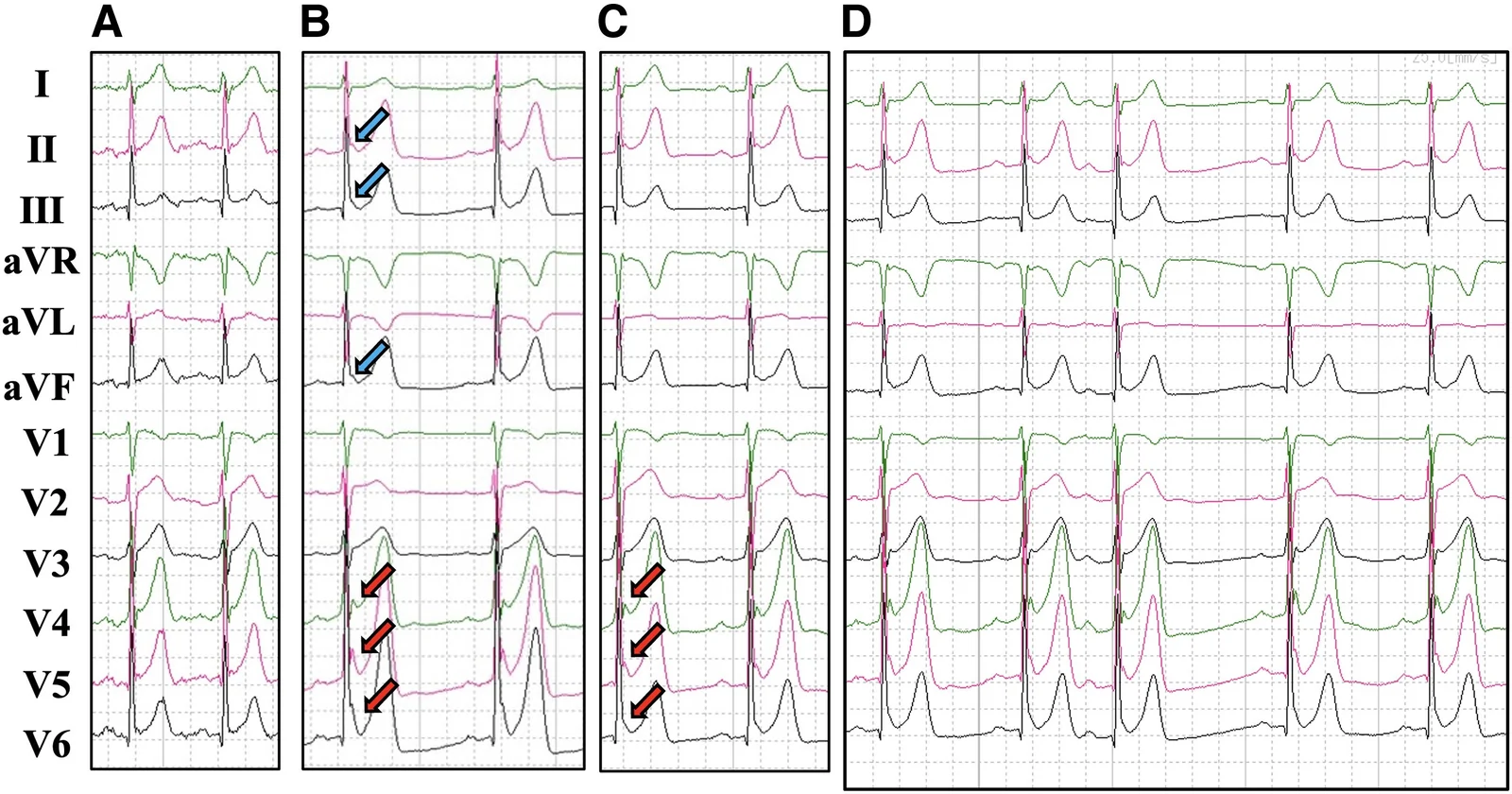
Changes in J-wave detected by 12-lead Holter electrocardiogram. (*A*) Holter electrocardiogram (baseline recording period) showed no significant J-waves. (*B*) At 4:15 a.m., electrocardiogram demonstrated prominent J-wave augmentation in leads V4–V6, with newly apparent subtle J-waves in inferior leads. (*C*) AT 5:45 a.m., electrocardiogram revealed progressive J-wave augmentation in both lateral and inferior leads, suggesting circadian rhythm-associated dynamic changes. (*D*) J-wave was enhanced after the atrial premature beat.

**Figure 3:**
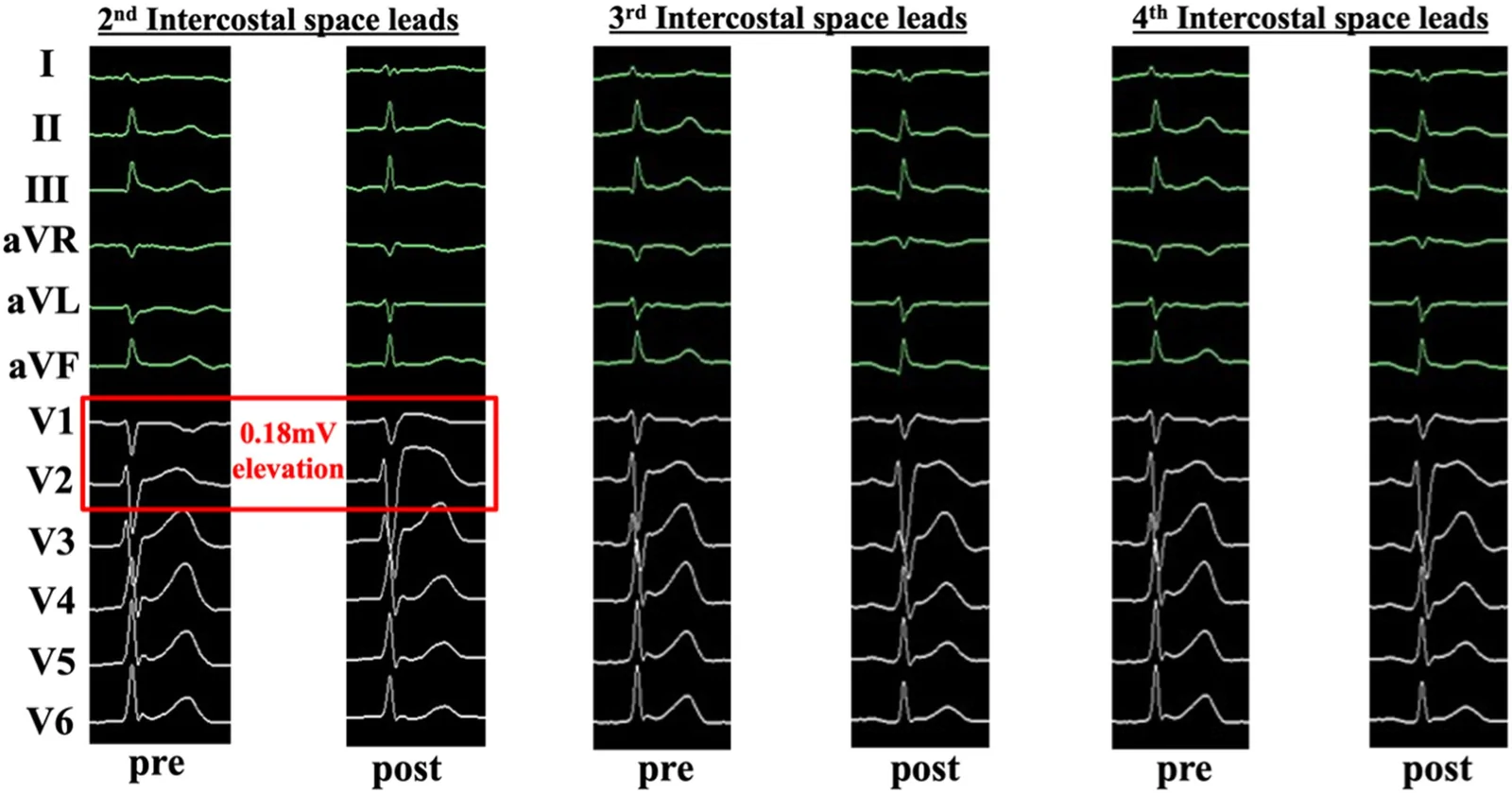
Pilsicainide loading test. Pilsicainide was administered intravenously at a rate of 1 mg/kg/h. The infusion was continued for 13 min. The 12-lead electrocardiogram following a pilsicainide challenge demonstrated a 0.18 mV J-point elevation in V1 at the second intercostal space, reduced J-wave amplitude in the inferior leads compared to baseline, and no significant change in the third or fourth intercostal spaces.

A 30-year-old man with no medical history, medication use, allergies, or family history of SCD experienced sudden syncope without prodromal symptoms while seated. He recovered spontaneously within 10 min with minor head trauma and urinary incontinence and presented to our hospital the same day. Vital signs, blood tests, and chest radiography were normal except for a temporal contusion. Brain magnetic resonance imaging showed no abnormalities. Electroencephalography was not performed; therefore, seizure activity, including epilepsy, could not be excluded. A 12-lead ECG showed elevated J-waves in V4–V6 without Brugada pattern or QT prolongation (*Figure 1*). Echocardiography demonstrated preserved systolic function without structural abnormalities. Because arrhythmic syncope was suspected, comprehensive cardiac evaluation was performed. Signal-averaged ECG showed no late potentials. Twelve-lead Holter monitoring demonstrated nocturnal J-wave augmentation in V4–V6 at 4:15 a.m. and 5:45 a.m. with subtle inferior J-waves (*Figure 2B* and *C*). No ventricular premature complexes were detected, and only two atrial premature complexes were observed. Coronary angiography showed no stenosis, and acetylcholine provocation testing was negative. Electrophysiological study revealed that atrium-to-His bundle interval and His bundle-to-ventricule intervals were 88 and 46 ms, respectively. Ventricular arrhythmias were not inducible during programmed stimulation, including rapid pacing and double extrastimuli at coupling interval (CI) of 240 and 200 ms. Effective refractory periods were 200 ms at the right ventricular apex and 190 ms at the right ventricular outflow tract. During a pilsicainide challenge test performed to unmask Brugada syndrome, a 0.18-mV J-point elevation was observed in V1 at the second intercostal space with attenuation of inferior J-waves (*Figure 3*). No changes were observed in the third or fourth intercostal spaces. Although early repolarization syndrome (ERS) was suspected, VF was not documented, and diagnostic criteria were not fulfilled. There was also little evidence of concealed cardiomyopathy, myocarditis, or arrhythmogenic cardiomyopathy; therefore, no additional testing was performed. The results of these tests are summarized in this table (*Table 1*).

**Table 1.** Summary of the diagnostic workup findings.

Test	Result
12-Lead ECG	Elevated J-waves in lead V4–V6
12-Lead Holter ECG	Dynamic J-wave augmentation was observed in lead V4–V6 at 4:15 and 5:45 a.m., with subtle J-waves in inferior leads
Signal-averaged ECG	No late potential
Pilsicainide loading test	0.18 mV J-point elevation in lead V1 (second intercostal space) with concomitant J-wave attenuation in the inferior leads
Electrophysiological study	No inducible VF or VT during programmed ventricular stimulation
Coronary angiography	No evidence of coronary artery stenosis
Acetylcholine provocation test	Negative

According to ESC guidelines, ICD implantation is recommended in patients with documented VF or haemodynamically intolerable VT without reversible causes.^4^ Because no structural heart disease or inducible arrhythmia was identified, the patient did not meet ICD criteria. However, given the syncopal episode and dynamic J-wave abnormalities, an ICM was implanted for remote monitoring. He was discharged without significant arrhythmias during administration.

**Figure 4:**
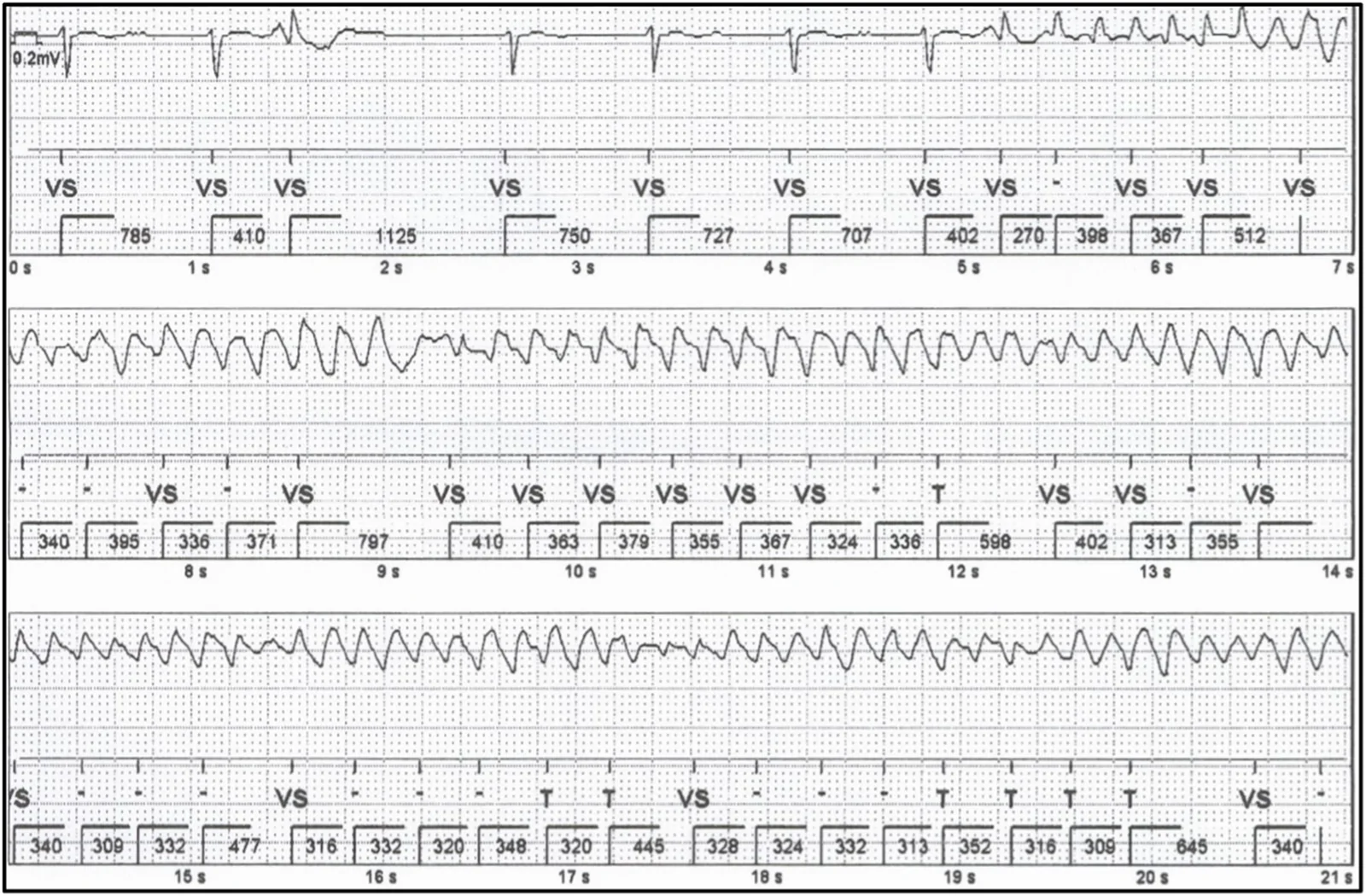
The insertable cardiac monitor recorded during the fatal event demonstrated a sequence of premature ventricular contractions with a 300-ms coupling interval, initiation of sustained ventricular fibrillation, and subsequent progression to sudden cardiac arrest.

Six months later, he developed convulsions while in a vehicle. Emergency medical services found him in cardiac arrest, and resuscitation was unsuccessful. Insertable cardiac monitor recordings demonstrated sustained VF initiated by a premature ventricular contraction with a 300-ms CI, confirming an arrhythmic cause of death (*Figure 4*). At this point, he was diagnosed with ERS.

## Discussion

Although most ER patterns are benign, risk stratification and prophylactic intervention remain challenging because high-risk cases are rare.^5^ This case demonstrates that patients classified as low risk by current ERS guidelines may still experience SCD, with the ICM providing definitive documentation of VF onset. These findings underscore the limitations of current risk stratification despite guideline-directed management. While conservative management was appropriate, fatal arrhythmias may occur even in ostensibly low-risk individuals. On the other hand, implanting an ICD in young patients poses important challenges, including the lifelong physical burden of repeated generator replacements and concerns about the cost-effectiveness of long-term follow-up and device management. However, cardiac MRI, autopsy, toxicology, genetic testing, and family screening were not performed, constituting a diagnostic limitation.

A previous study reported that 4.9% of ER patients with syncope developed VT/VF or SCD over 5 years.^6^ Features, including ER amplitude >0.2 mV, horizontal ST segments, and family history of SCD, did not predict VT/VF. However, another study linked non-type I anterior ER, J-wave abnormalities in V1–V3, to higher VF risk in lower-lateral-wall-type ERS.^7^ In the present case, pilsicainide challenge testing produced a 0.18-mV J-point elevation in V1. ‘The threshold of J-point elevation ≥0.2 mV’ is based on expert consensus, and its precise clinical significance remains uncertain.^8^ Even if considered positive, drug-induced Type 1 ECG with syncope alone is not an established indication for ICD implantation.^4^

Implantable cardioverter-defibrillator implantation is clearly indicated in patients with prior cardiac arrest or documented sustained ventricular arrhythmias, whereas management of syncope depends on the likelihood of an arrhythmic mechanism and the overall clinical context. Therefore, careful characterization of syncope and individualized risk assessment are essential.

Twelve-lead Holter monitoring revealed dynamic nocturnal J-wave augmentation. Previous studies have shown a temporal association between J-wave augmentation and VF storms in ERS patients, although its role as an independent predictor of future VF remains unproven.^9^ Additionally, J-wave enhancement following an atrial premature beat was observed (*Figure 2D*), a finding associated with electrical vulnerability after long cycle lengths.^10^ Although insufficient to justify ICD implantation, these findings may contribute to future risk stratification.

Another report states that among ERS patients with a history of VF, those who have two or more of the following three factors—‘QRS abnormalities (wide QRS or fQRS)’, ‘wide J-wave’, and ‘abnormal T/R ratio’—are at high risk for VF.^11^ ‘Wide QRS’ was defined as QRS width ≥100 ms, while ‘fQRS’ was defined as an additional R wave or multiple R′ waves in the descending portion of the R or S wave and was considered positive in ≥3 leads. A J-wave width ≥57 ms was considered high risk, and a ‘high T/R ratio’ was defined as an R-to-T wave ratio ≥0.42 in Lead II. In this case, QRS width was 80 ms, fQRS was observed only in Lead III, J-wave width was 68 ms, and the T/R ratio in Lead II was 0.55. These findings suggest that the present case could have been identified as high risk for VF. However, since this previous report focuses on cases with a history of VF, it may not be applicable to the present case.

This case raises the question of whether alternative preventive measures could have prevented the death. Strategies such as educating family members and co-workers, emergency response planning, and workplace automated external defibrillator (AED) installation may be valuable when ICD implantation is not indicated. A Belgian study reported lives saved by workplace AEDs but emphasized the need for standardized implementation and training.^12^ Individualized risk communication is therefore essential in ERS patients with subtle risk markers.

This rare case of ICM-confirmed SCD in ERS underscores the limitations of current risk models. Retrospective review demonstrated dynamic ECG changes, including nocturnal J-wave augmentation and pilsicainide-induced J-point elevation, which may help identify patients at increased arrhythmic risk. The case also highlights the value of longitudinal ECG surveillance and reassessment of evolving electrocardiographic findings, even in patients initially considered low risk.

## Patient’s perspective

This case had the limitation that the patient perspective could not be obtained because of the fatal outcome.

## Lead author biography


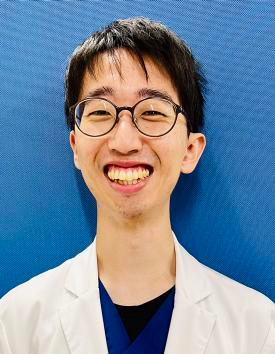


Dr Soichiro Yamashita is a 2024 Medical Doctor in Awaji Medical Center.

## Data Availability

The authors confirm that written consent for submission and publication of this case report including the images and associated text have been obtained from the patient in line with COPE guidance.

## Notes

**Consent:** Informed written patient consent has been obtained, in line with the COPE best practice guidelines, and the individual who is being reported is aware of the possible consequences of that reporting.
